# Laparoscopic approach to refractory extraspinal sciatica and pudendal pain caused by intrapelvic nerve entrapment

**DOI:** 10.1038/s41598-021-90319-y

**Published:** 2021-05-24

**Authors:** Nucelio Lemos, Corey Sermer, Gustavo Fernandes, Augusta Morgado-Ribeiro, Andrea Rossos, Zi Ying Zhao, Manuel J. B. C. Girão, Philip Peng

**Affiliations:** 1 Department of Obstetrics and Gynecology, Mount Sinai and Women’s College Hospital, University of Toronto, Toronto, ON Canada; 2 Department of Gynecology, Federal University of São Paulo, São Paulo, Brazil; 3 Department of Anesthesiology and Pain Management, Mount Sinai and Women’s College Hospital, University of Toronto, Toronto, ON Canada; 4 Department of Obstetrics and Gynecology, Mount Sinai and Women’s College Hospital, University of Toronto, 700 University Avenue|Room 8-917, Toronto, ON M5S 1Z5 Canada

**Keywords:** Medical research, Neurological disorders

## Abstract

Entrapments of the intrapelvic portions of the lumbosacral plexus are an important extraspinal cause of sciatica and pudendal neuralgia. They can be treated using Laparoscopic Neuronavigation (LANN), a minimally invasive technique that has set the foundations of an emerging field in Medicine—Neuropelveology. This retrospective-prospective study analyzes the outcomes of 63 patients treated with the LANN technique over a 10 year time period. One year after surgery, 78.3% of patients reported clinically relevant pain reduction, defined as ≥ 50% reduction in Numeric Rating Scale (NRS) score; these results were maintained for a mean follow up of 3.2 years. Preoperative chronic opioid use (≥ 4 months of ≥ 10 mg morphine equivalents/day) was a predictor of poor surgical outcome—clinically relevant pain reduction was observed in only 30.8% in this group of patients, compared to 91.5% in patients not regularly taking opioids preoperatively (p < 0.01). Perioperative complication rate was 20%. Our results indicate that the LANN technique is an effective and reproducible approach to relieve pain secondary to intrapelvic nerve entrapments and that preoperative chronic opioid therapy significantly reduces the likelihood of a successful surgical outcome. This study provides detailed information on perioperative complication and postoperative course, which is essential for patient consenting.

## Introduction

Although usually attributed to spinal, deep gluteal and/or perineal causes, sciatica and pudendal neuralgia can result from entrapments of the intrapelvic portion of the lumbosacral plexus. As with nerve entrapment syndromes in other parts of the body, intrapelvic nerve entrapments (INEs) are characterized by pain and dysfunction of the lumbosacral nerves’ dermatomes and myotomes, causing pain in the lower limb (sciatica), perineum and gluteal regions, lower urinary tract symptoms, rectovesical tenesmus, dyschezia, vaginal foreign body sensation, dysuria, dyspareunia and/or painful ejaculation^1–6^.

INEs were largely unknown before the development of the Laparosocopic Neuronavigation (LANN) technique, by Possover et al.^7^. The LANN technique introduced a systematic and minimally invasive approach to dissect, expose and detrap the intrapelvic nerves, opening the possibility for diagnostic confirmation and treatment of INEs. Additionally, this technique has led to a better understanding of entrapment neuropathies and other causes of dysfunction of the intrapelvic nerves, which resulted in the development a new transitional medical field—Neuropelveology^8^. Possover et al. proposed a ground-breaking Neuropelveological rationale^8^, which allows for the diagnosis and management of intrapelvic causes of pudendal neuralgia and sciatica resulting from entrapments at the origin of nerve roots from the sacral foramina down to the sciatic notch and pudendal canal, with success rates close to 80%^9^. While extremely encouraging, these results are based on narrative case series and their reproducibility still needs to be demonstrated by other groups.

Since late 2009, our team has been offering laparoscopic nerve detrapment procedures using the LANN technique in São Paulo, Brazil. The objective of this study is to report the clinical outcomes of this procedure in our cohort of patients from December 2009 to December 2019.

## Methods

This is a retrospective-prospective analysis of 63 consecutive patients who underwent a LANN detrapment of intrapelvic portions of the lumbosacral plexus from December 2009 to December 2019 at our Neuropelveology center in São Paulo, Brazil.

Patients were identified from our surgical database. All the surgical reports within the study period were reviewed and all the patients who underwent LANN detrapment surgery were included. Data was retrospectively extracted from their electronic medical records until 2018, when this research protocol was approved by the research ethics board; data has been prospectively collected since. All data was extracted into a Microsoft Excel database file which began to be prospectively updated in 2018.

The diagnosis of INEs was based on Neuropelveological assessment, following the standards of the International School of Neuropelveology^10^. This comprehensive assessment included a full Neuropelveology-oriented history, neurological examination, urodynamic study, pelvic MRI and a diagnostic nerve block.

Pain was assessed with a Numeric Rating Scale (NRS), with 0 representing no pain and 10 the worst imaginable pain, at the pre-operative and all subsequent post-operative follow-up visits. Clinically relevant pain reduction was defined as a ≥ 50% reduction in NRS. It is widely accepted in the pain literature that a minimum reduction of 30% or 2-point reduction in a numeric pain scale (from 0–10) defines a clinically meaningful improvement in pain^11^. However, it is common for pain relief to deteriorate over time and therefore a higher cut off (50%) is often used for longer-term follow-up studies^12^.

The primary outcome of this study was clinically relevant pain reduction at 1 year follow up. Data from the patient’s most recent follow up was also extracted and analyzed.

In addition, the following pre-operative and intra-operative information was collected from the patients’ records: the time of the onset of symptoms, duration and dose of preoperative opioid use, entrapment location and etiology, operative time, intraoperative blood loss and perioperative complications. From this information, the diagnostic lag was calculated and was defined as the time between the onset of symptoms and the time of the LANN detrapment surgery. The operative time was determined from video recordings, defined as the moment the laparoscopic camera was inserted into the abdomen to the moment all ports were removed.

Post-operative complications were also extracted from follow-up visits notes. Post-decompression neuralgia (PDN) was defined as pain on the same dermatomes where pain was experienced pre-operatively, with different quality and/or intensity. Any motor deficit within the lumbosacral plexus myotomes developed after surgery was also identified and collected. In the subsequent follow up appointments, the duration of PDN and motor deficits were recorded.

### Statistical analysis

Extracted data was put into an MS-Excel database and analyzed in Prism (version 6.0, GraphPad, San Diego, CA, USA).

A chi-squared test was used to assess the proportions of clinically relevant pain reduction rates at 1 year and at final follow-up, to ensure maintenance of pain reduction.

To account for patients whose one-year follow up data was not available, three different sub-analyses were performed: patients with missing data excluded, patients with missing data considered a failure (worst case scenario analysis) and considering the last follow up data to be the 1-year follow up data (carry-over analysis).

A Komolgorov-Smirnov test was used to assess the normality distribution for NRS scores, which showed that this data did not follow a Gaussian distribution. As such, non-parametric tests were used in our analyses. A paired Wilcoxon’s test was used to compare preoperative and 1-year follow up NRS scores. For this paired test, patients with no 1-year follow up data were excluded from the 1-year analysis. A paired Wilcoxon’s test was also used to compare preoperative NRS score with the NRS score of the last follow up. Finally, to determine if results were unchanged over time, a Wilcoxon’s test was used to compare 1-year NRS scores with the scores from the last follow up.

A chi-squared test was used to determine if preoperative chronic opioid use—defined as daily use ≥ 10 mg morphine equivalent for at least 4 months—or if a diagnostic lag of at least two years affected rates of clinically relevant pain reduction. A Mann–Whitney test was used to compare pre-operative NRS homogeneity of both subgroups. Descriptive statistics were used for the remainder of the data.

### Ethical proceedings

This study was approved by the Research Ethics Board of the Federal University of São Paulo (Protocol # 3.272.139). Consent for collection and storage of images and clinical data for quality improvement, medical education, scientific studies and publications was obtained from all subjects, as a standard practice in surgical consenting process. This consent for authorization of usage of clinical data and images was approved by the REB as part of the ethical analysis. All the proceedings and methods of this study are in accordance with Brazilian research ethics regulations and the Declaration of Helsinki.

This study was also registered at the Brazilian Clinical Trials Registry (UTN code: U1111-1261-4910).

## Results

A total of 63 patients (56 females and 7 males) underwent laparoscopic nerve root decompression during the study inclusion period. The average age of the patients included in this study was 39.9 ± 10.6 years (Table 1).

**Table 1:** Patient demographics and surgical outcomes.

	Mean	Median	Standard deviation
Age (years)	39.7	37.8	10.5
Follow-up (years)	3.0	2.5	1.8
Pre-op NRS	8.7	9.0	1.8
Post-op NRS at 1 year post-op	2.7*	2.0	2.9
Post-op NRS at final follow-up	2.9*	2.0	3.2
Operating time (min)	167.5	144	87.8
Previous surgeries	1.1	1.0	1.4
Diagnostic lag (years)	4.5	3.0	4.1

*Paired Wilcoxon’s test comparing pre-op NRS and post-op NRS at 1 year and final follow-up (p < 0.001).

Two patients were lost to follow-up. Additionally, there was one patient that had surgery within less than one-year of publication, and as such had not yet had a 1-year follow-up appointment. Therefore, one-year follow up data was available for 95.2% (60/63) of patients.

Clinically relevant pain reduction, defined as a ≥ 50% improvement in NRS for pain, was 78.3% (47/60) 1 year after surgery (p < 0.001; Chi-squared test). This improvement in pain scores was maintained through the patient’s final follow-up appointment (73.3% (44/60); p = 0.670, Chi-squared test, for one-year versus last follow up), at an average of 3.2 ± 1.8 years. The mean NRS dropped significantly from 8.7 ± 1.8 pre-operatively, to 2.7 ± 2.9 at the 1-year follow-up (p < 0.001), and 3.0 ± 3.2 at final follow-up (p < 0.001). NRS did not significantly differ from the 1 year follow-up to last follow up (p = 0.0502; paired Wilcoxon test) time points, suggesting outcomes are maintained over time (Table 1).

When patients with missing data were excluded, clinically significant pain reduction was achieved on 47/60 (78.3%) of patients. No statistically significant difference in results were observed when patients with missing data were considered failures (*worst case scenario* analysis, 74.6% versus 78.3%, p = 0.6749) or when the result from the last follow up was carried over to one-year follow up (carry over analysis, 79.4% versus 78.3%, p = 0.888).

Preoperatively, 13/60 patients (21.7%) consumed ≥ 10 mg morphine equivalents daily, for an average of 18.6 ± 9.6 months. Pre-operative chronic opioid use adversely affected the primary outcome, as the clinically relevant pain reduction was observed in only 30.8% (4/13) in this group of patients, compared to a 91.5% (43/47) in patients not taking opioid on a regular basis (p < 0.01). Preoperative NRS scores were not significantly different between the two subsets of patients—8.6 (± 1.8; 3–10) for non-opiod vs. 9.0 (± 1.7; 5–10) for opioid users (p = 0.4458). The mean time interval between onset of symptoms and surgery was 4.9 ± 4.3 (0.3–15) years for opioid users and 4.6 ± 4.0 (0–15) years for non-opioid users. This difference was not statistically significant (p = 0.7902).

The average interval between symptom onset and surgery was 4.73 ± 4.1 years, with patients having undergone an average of 1.1 ± 1.5 years surgical procedures for treatment of their symptoms prior to our LANN detrapment (Table 1). Three out of 21 (14.3%) patients had unsuccessful outcomes when surgery was performed within 2 years of symptom onset compared to 10/39 (25.6%) when surgery was performed after 2 years from symptom onset, however this was not statistically significant (p = 0.5461). Preoperative NRS were also homogeneous between the two groups (p = 0.594). The time interval between onset of symptoms and surgery was 4.6 ± 4.0 (0–15) years for patients with clinically significant pain reduction and 4.8 ± 4.3 (0.5–15) years for patients in whom clinically significant pain reduction was not achieved. This difference was not statistically significant (p = 0.8308).

**Figure 1:**
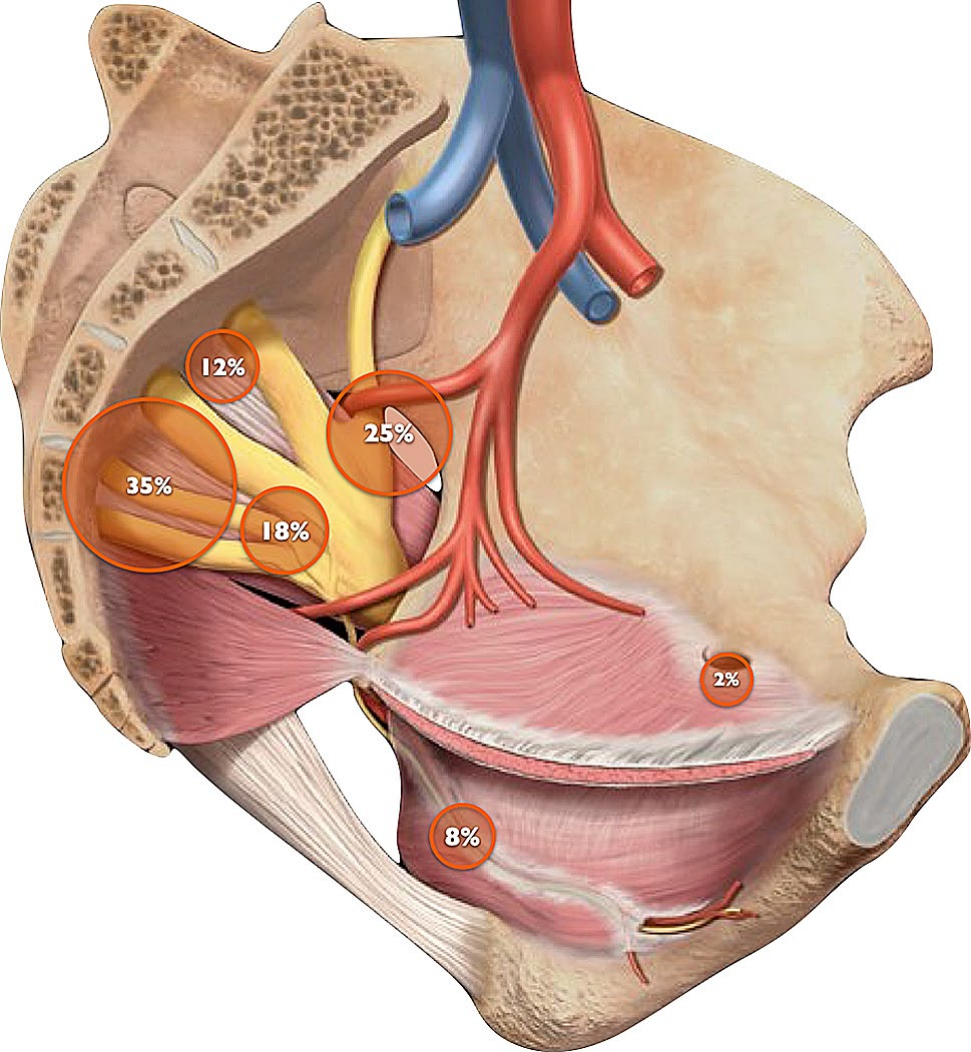
Distribution of nerve entrapments locations: 35% in proximal S2/S3/S4 nerve roots, 25% in sciatic/lumbosacral, 18% in proximal pudendal/medial sciatic, 12% in S1/S2 nerve roots, 8% in Alcock’s canal level and 2% in obturator nerve entrapment.

Distribution of nerve entrapments locations are displayed in Fig. 1. The most common entrapment site was proximal S2/S3/S4 nerve roots, in 21 (35%) cases, followed by lateral sciatic/lumbosacral trunk in 15 (25%), proximal pudendal/medial sciatic in 11 (18%) and S1/S2 nerve roots in 7 (12%). Entrapments at the Alcock’s canal level were present in only 5 (8%) patients and one patient had an obturator nerve entrapment, caused by a transobturator sling.

**Figure 2:**
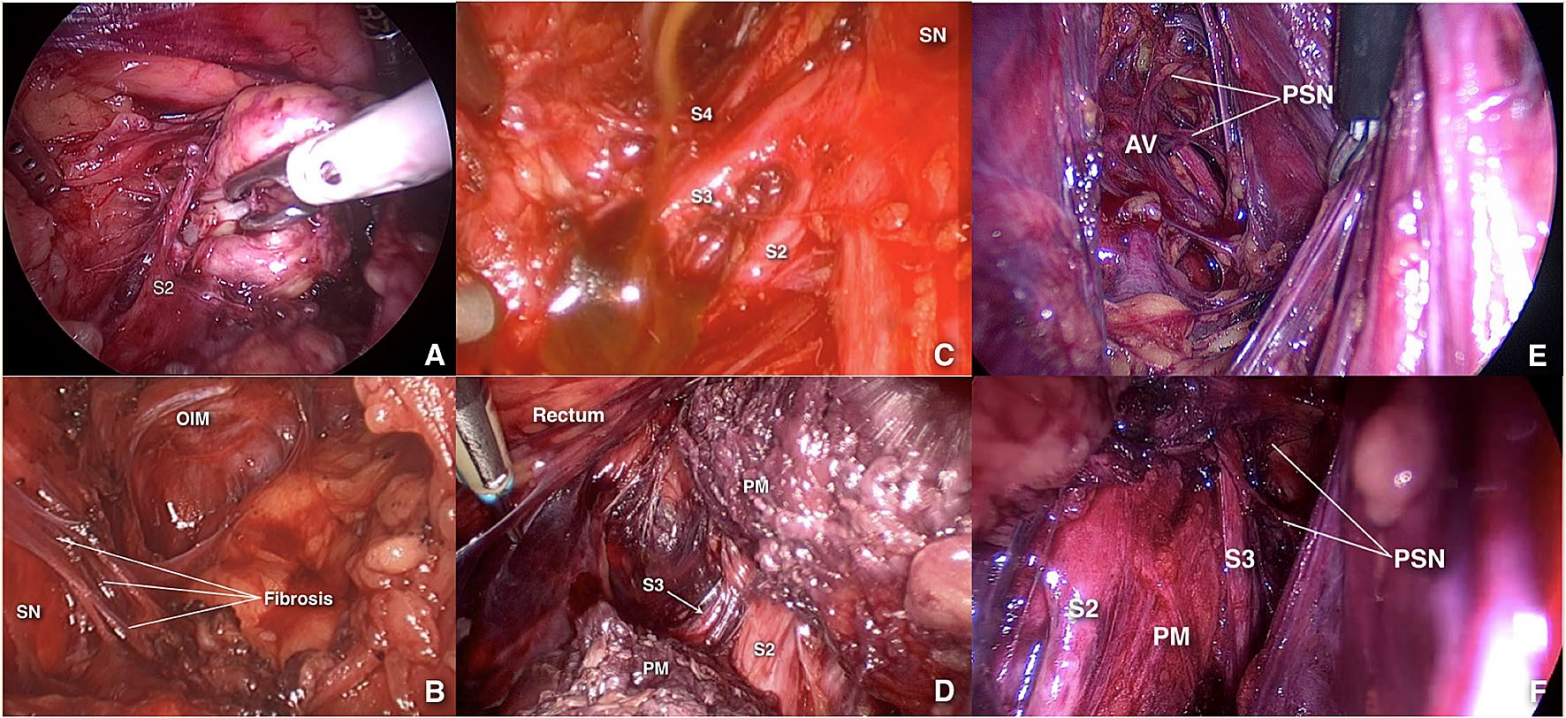
The five etiologies of intrapelvic nerve entrapment: neoplastic (**A**), fibrotic (**B**), endometriotic (**C**), abnormal piriformis muscle bundle originating medially to the sacral foramina (**D**) and neurovascular conflict before (**E**) and after (**F**) decompression. *SN* sciatic nerve; *PM* pyriformis muscle; *OIM* obturator internus muscle; *PSN* pelvic splanchnic nerves; *AV* abnormal vein.

The five etiologies of nerve entrapments identified during surgery in these 60 patients were endometriosis, fibrosis, piriformis entrapment by abnormal muscle fibres originating medial to the sacral foramina, neurovascular conflict, and neoplasm (Fig. 2). Neurovascular conflict and endometriosis were the most common etiologies of entrapment in this group of patients (38.3% and 36.7%, respectively), followed by fibrosis (10%), abnormal piriformis bundles originating medially to the sacral foramina (10%), and nerve sheath tumors (1.7%). Two patients had both fibrosis and abnormal piriformis muscle bundles causing their INE (3.3%).

The average operating time was 168 ± 89.2 min (Table 1). Perioperative complications included 1 pudendal nerve transection, 1 obturator nerve tear, 1 ureteral injury, 2 cases of genitofemoral neuropathy, 1 incisional hernia, 1 rectovaginal fistula, and 1 case of piriformis muscle adhesion to the sciatic nerve requiring a transgluteal endoscopic procedure. One patient required a blood transfusion, 2 patients had transient urinary retention and 1 patient had impaired bladder sensation. Overall, 20% (12/60) of patients had a perioperative complication; of these, the rectovaginal fistula and the ureteric injury were attributable to endometriosis treatment, not the nerve detrapment per se, resulting in a LANN detrapment complication rate of 16.7%.

During the postoperative course, 56.7% (34/60) of patients experienced post-decompression neuralgia, lasting on average 5.7 ± 3.4 (1–12) months. Fourteen (23.3%) patients experienced a post-decompression motor deficit, lasting on average 3.2 ± 3.2 (0.5–12) months.

## Discussion

It is well known that a large portion of the lumbosacral plexus is located intra-abdominally, in the retroperitoneal space^13^. However, most literature descriptions of lesions on this plexus refer to its extra-abdominal parts, in the spine or deep gluteal space, while reports of intrapelvic entrapments are traditionally limited to isolated case reports.

Currently, intrapelvic entrapments are not well understood and rarely considered as a cause of sciatica and pudendal neuralgia. One of the reasons for this is the deep retroperitoneal location of the plexus, in an area that can only be adequately approached by laparoscopy, which provides better lighting, magnification and visualization of this area. Therefore, since the specialists that usually deal with peripheral nerve surgery (neurosurgeons, orthopedic and plastic surgeons) are not usually trained in laparoscopy and those specialists who routinely perform advanced laparoscopic procedures (gynecologists, urologists and gastrointestinal surgeons) are not familiarized with peripheral nerve surgery, a knowledge gap is created^2^. Therefore, none of the traditional and widely available residency and fellowship programs offers training in the skills set required for the diagnosis and management of INEs.

In 2004, Possover et al. pioneered the Laparoscopic Neuronavigation (LANN) technique^7^, opening the doors to accessing the intrapelvic portion of the lumbosacral plexus through a safe and minimally invasive way. This led to the development of a new groundbreaking medical field—Neuropelveology^8^.

The LANN technique was initially developed to standardize nerve-sparing approaches in retroperitoneal dissection during cervical cancer or deeply infiltrating endometriosis surgeries^14^. The LANN technique was later proposed as a minimally invasive detrapment method to significantly reduce or completely resolve pain secondary to INEs. This surgical intervention follows the same principles of surgeries that have been successfully used in the treatment of other peripheral nerve entrapment syndromes^9,14,15^.

In 2009, a series of 134 patients who underwent laparoscopic nerve decompression for refractory anogenital pain showed improvement in VAS scores depending on etiology, with significant decrease in pain achieved in 83% of patients with Alcock’s canal syndrome, 62% of patients with endopelvic lesions, and 78% of patients with endometriosis^9^. Additionally, a study involving 120 patients showed that 65.5% of patients experienced significant improvement in somatic pelvic pain following laparoscopic nerve decompression^6^. Recently, the same group reported similar outcomes in a series of 213 female patients with lumbosacral entrapment due to neurovascular conflict and/or endometriosis^1^.

This is the first case series in the literature showing the reproducibility of the Neuropelveological approach proposed by Possover et al.^9^ for INEs. It is also the first study with a systematic assessment, as previous reports of INEs are generally narrative case series. In our study, we had a clear time point at one-year follow up for the primary outcome. In addition, this is the first series to report the sustainability of such outcomes at mid to long-term follow up of 3.2 years, on average.

This is also the first report to show the negative impact chronic opioid use has on long-term outcomes following LANN detrapment of intrapelvic nerves. We show here that chronic opioid use reduces the odds of success of this etiology-oriented treatment from 91.5% to 30.8%. This is of extreme importance as it supports the concept that chronic exposure to opioids may predispose a patient to opioid induced hyperalgesia, thus resulting in a lower pain threshold and thus poor analgesic outcomes^16,17^. Prolonged preoperative opioid exposure was the only predictor for poor surgical outcome identified in our cohort; preoperative pain scores and time between onset of symptoms and surgery, two factors we considered would be associated with these results, were homogeneous between chronic opioid users and opioid naïve patients. Such information should influence opioid prescription habits and motivate the use of this medication class only as end-stage therapy. Alternatively, the use of multimodal non-opioid pain management strategies should be promoted in patients with all other forms of chronic pain, as opioids might jeopardize their chances of cure and make them prone to lifelong pain.

We have also described the different locations of entrapments, which is very important for understanding the dermatomes and myotomes implicated in INEs. It is important to highlight the very small proportion of entrapments at the level of Alcock’s canal (8%) observed in our series. This is very significant, given that “pudendal neuralgia” is often used interchangeably in the literature with “Alcock’s Canal Syndrome”. Also, the most common criteria for diagnosis of pudendal nerve entrapments^16^ was developed to diagnose entrapments at the level of Alcock’s Canal and/or at the interligamentous plane. The fact that the most common site of pudendal entrapments in our cohort are the pudendal nerve roots could possibly explain several cases of “atypical pudendal neuralgia” or surgical failures seen in the literature. For the purpose of differential diagnosis, we should raise awareness for the combined symptoms of pudendal pain and sciatica and/or gluteal pain, which could be explained by an INE at the level of the sacral nerve roots.

Another important finding in our report is the long diagnostic lag experienced by our patients, who have suffered for 4.7 years and undergone 1.1 previous failed surgeries in an attempt to treat their symptoms. This further illustrates the knowledge gap and lack of awareness of INEs as an extraspinal cause of sciatica and pudendal pain. This data is also consistent with the data reported on European cohorts, in whom an average diagnostic gap of 5.6 years was documented and an average of 4 surgical procedures were conducted before an INE was suspected^6^.

On top of the prolonged suffering, the long diagnostic gap can have other consequences. First, the success rates of detrapment procedures would be adversely affected^18^. In our patients, the failure rate of LANN decompression was 86% higher when surgery was performed more than 2 years from symptom onset. Although this difference in failure rate did not reach statistical significance, likely due to our limited sample size, it is consistent with the current literature, meaning this likely reflects a reality^4,15,19–21^.

Secondly, lack of awareness of INEs increases the risk of patients undergoing ineffective surgeries which do not address the underlying cause of pain and may cause added harm. Moreover, each surgical attempt further prolongs the treatment delay, as patient and surgeon will need time to assess surgical outcomes. Patients who have been waiting for a long time tend to catastrophize^22,23^; under pressure, healthcare professionals may resort to the use of opioids^24^. This is of particular importance, as we have shown here that chronic opioid use decreases the chances of a successful surgical outcome.

In this study we have also systematically assessed the postoperative course. Approximately half of our patients experienced post-decompression neuralgia, which lasted on average about 6 months and almost a quarter of patients experienced a post-decompression motor deficit, lasting approximately 3 months. It is important to make patients aware that post-decompression neuralgia and motor deficits are part of the natural postoperative course, since, if long lasting, they can be misinterpreted as permanent and cause significant distress.

In addition, the perioperative complication rate was 20% in our cohort, ranging from nerve injuries to transient bladder symptoms including retention and impaired sensation. For intricate surgery on peripheral nerves, such as LANN detrapment, this complication rate is within the acceptable range, but discussing these risks with patients is essential for appropriate preoperative counselling and consenting.

This current study is limited by the relatively small cohort, retrospective design and the use of NRS as the singular outcome measure. However, the consistency with Possover et al.’s^13^ results, as well as the similarity of the outcomes with surgical treatment of other nerve entrapment syndromes suggests that our results can be extrapolated to a larger population.

Future studies assessing the impact of laparoscopic nerve detrapment surgery on different variables, such as sexual function, quality of pain, bowel/bladder dysfunction, and psychosocial components of chronic pain, will help to create a more robust understanding of the outcomes of this approach. Additionally, following patients for a longer post-operatively will result in further understanding of the maintenance of the effects of surgery and the future need for additional pain management medications or procedures.

Nevertheless, our results validate the Neuropelveology approach to diagnose and treat INEs and highlight the importance of raising awareness of the risk of non-specific pain management opioids, which significantly reduces the likelihood of a successful surgical outcome.
